# An eHealth Intervention to Promote Physical Activity and Social Network of Single, Chronically Impaired Older Adults: Adaptation of an Existing Intervention Using Intervention Mapping

**DOI:** 10.2196/resprot.8093

**Published:** 2017-11-23

**Authors:** Janet M Boekhout, Denise A Peels, Brenda AJ Berendsen, Catherine AW Bolman, Lilian Lechner

**Affiliations:** ^1^ Faculty of Psychology and Educational Science Open University of the Netherlands Heerlen Netherlands

**Keywords:** exercise, older adults, single, chronic disease, eHealth, social network, Intervention Mapping

## Abstract

**Background:**

Especially for single older adults with chronic diseases, physical inactivity and a poor social network are regarded as serious threats to their health and independence. The Active Plus intervention is an automated computer-tailored eHealth intervention that has been proven effective to promote physical activity (PA) in the general population of adults older than 50 years.

**Objective:**

The aim of this study was to report on the methods and results of the systematic adaptation of Active Plus to the wishes and needs of the subgroup of single people older than 65 years who have one or more chronic diseases, as this specific target population may encounter specific challenges regarding PA and social network.

**Methods:**

The Intervention Mapping (IM) protocol was used to systematically adapt the existing intervention to optimally suit this specific target population. A literature study was performed, and quantitative as well as qualitative data were derived from health care professionals (by questionnaires, n=10) and the target population (by focus group interviews, n=14), which were then systematically integrated into the adapted intervention.

**Results:**

As the health problems and the targeted behavior are largely the same in the original and adapted intervention, the outcome of the needs assessment was that the performance objectives remained the same. As found in the literature study and in data derived from health professionals and focus groups, the relative importance and operationalization of the relevant psychosocial determinants related to these objectives are different from the original intervention, resulting in a refinement of the change objectives to optimally fit the specific target population. This refinement also resulted in changes in the practical applications, program components, intervention materials, and the evaluation and implementation strategy for the subgroup of single, chronically impaired older adults.

**Conclusions:**

This study demonstrates that the adaptation of an existing intervention is an intensive process in which adopting the IM protocol is an invaluable tool. The study provides a broad insight in adapting interventions aimed at single older adults with a chronic disease. It is concluded that even when the new target population is a sizable segment of the original target population, the adapted intervention still needs considerable changes to optimally fit the needs and situational differences of the narrower target population.

## Introduction

The international guideline for physical activity (PA) recommends that one should be physically active with moderate to vigorous intensity for at least 5 days a week with a minimum of 30 min a day [1]. Regular PA reduces the risks of multiple health problems, such as chronic diseases. For individuals with preexisting health problems, being sufficiently physically active may be accompanied with more barriers but can still result in an improved health and in a reduced risk of developing comorbidities [1,2]. Especially for older adults (people older than 65 years), the health benefits of sufficient PA are relevant as PA also prevents cognitive decline [3] and improves balance, thus decreasing the risk of fall-related injuries [4,5]. Moreover, PA is beneficial for mental health, as it reduces stress and depression and has positive outcomes on general well-being, such as an increased social connectedness [2,6].

Despite these benefits, PA decreases with age. In the Western world, 19% of the younger adults do not meet the recommended level of PA, compared with 55% of the older adults [7]. In certain subpopulations, a lack of sufficient PA is even more prevalent. In general, people with a chronic disease are less physically active than healthy people of their age group. In the Netherlands, 42% to 71% of the chronically ill people who are older than 55 years meet the recommended level of PA, whereas 84% of the healthy people in this age group meet the recommended level [8]. Western society in general shows a similar image [9]. Chronic diseases often come with impairments to PA [10,11]. The lack of PA causes the physical condition to increasingly deteriorate and daily activities to cost more energy, ultimately resulting in a downward spiral [12-14]. Another significant feature of older adults, who are less physically active, is not having a life partner [6,15]. In addition to their lower level of PA, older people who do not have a life partner are at risk for becoming socially isolated, which often results in feelings of loneliness. Loneliness is considered to be a significant risk factor for negatively affecting the physical and mental health of single older adults [16,17]. Promotion of PA, preferably done with others, in single older adults with a chronic disease is not only important from a physical and mental health perspective but also to stimulate participation in social life and society [18-20]. Several studies have shown reciprocal relations between PA and quality of life [21-23]. No international data are available on what part of the population is single, as well as older than 65 years and has a chronic disease. Available data in the Netherlands suggest that this group is a large proportion of the total population: in the age group of 65 to 84 years, the percentage of people with a chronic disease (with or without physical impairments) ranges between 78% and 93%; of the people older than 65 years, 39% are single [24]. It is assumed that the distribution of these characteristics is not very divergent from other Western countries. Stimulating PA is, therefore, of major relevance in this group, not only because of the benefits PA has for this group but also because of its proportion.

Previously, the computer-tailored Active Plus intervention (hereafter named Active Plus50) was developed to increase PA among people older than 50 years [25,26]. The intervention is available with a printed or a Web-based delivery mode. Active Plus50 provides tailored advice 3 times: after the first questionnaire at baseline, after 2 months, and after the second questionnaire (3 months after baseline). Although Active Plus50 was effective in stimulating PA in its target population (ie, people older than 50 years) [27,28], as well as in its subpopulation of single older adults (older than 65 years) with a chronic disease that impaired them in their PA behavior, this particular subpopulation often felt the intervention was not sufficiently adapted to their specific needs and expectations with regard to PA [25]. Furthermore, this subpopulation also indicated that even when they are stimulated to be more physically active, they often lack knowledge about their possibilities to be physically active in their own surroundings, given their impairment. Moreover, results of the qualitative evaluation showed that this subpopulation would like more opportunities for personal and social contacts in the program. With these findings, it became apparent that the intervention could remain as it was for the general target population of people older than 50 years, but that it needed adaptation for the specific subpopulation of single older adults with a chronic impairment in PA. This study describes how the proven effective, evidence-based Active Plus50 intervention, aimed at raising and maintaining the amount of PA for people older than 50 years, was systematically adapted to better fit the needs and characteristics of older adults who are single and have one or multiple chronic physical impairments. To increase the chance that the adapted intervention (hereafter named Active Plus65) will remain effective, the Intervention Mapping (IM) protocol was used [29]. This study adds to the current knowledge on adaptation processes for existing interventions—a broad insight in adapting existing interventions was established by combining theory, previous research, and input from the target population and health care professionals.

## Methods

### Intervention Mapping Steps for Modifying an Intervention

When an evidence-based intervention is modified, it may lose its effectiveness. Therefore, it is essential to carefully consider which components of the intervention are crucial to its effectiveness and, therefore, cannot be modified, and which elements can be modified without changing the effectiveness substantially. To develop new interventions and modify existing ones, several models and protocols have been developed. A well-studied and often used protocol is the IM protocol [29]. Compared with other models and protocols, IM enables the developer of the intervention to take all the necessary steps to develop or modify an intervention. The IM protocol for modifying an existing intervention differs from the IM protocol for developing new interventions. There are also 6 consecutive steps, but the content of the steps is different from that of the development model. The basic content of these steps, and how they are executed specifically in this study, are stated below.

#### Step 1: Needs Assessment and Determination of Fit With the Problem

In this step, a logic model for the problem is constructed, that is, the problems for the target group regarding health, quality of life, related behavior, and environment are assessed. Step 1 should result in a description of discrepancies between the problems as they are seen in the existing intervention and in the adapted intervention for the new (or in this study, a specific part of the original) high-risk target population. A literature study was conducted to achieve this goal in adapting Active Plus50. Furthermore, inputs from process evaluations from previous studies on Active Plus50 were used.

#### Step 2: Defining a Logic Model of Change and Matrices

This step aims to determine whether performance objectives, behavioral determinants, and change objectives of the original intervention need to be modified to better fit the new target population. The original performance objectives resulting from the development of Active Plus50 [26] are shown in Table 1.

Performance objectives consist of behavior that the target population has to perform to reach the program objective. The performance objectives can be regarded as specifications of the program objective, that is, the ultimate goal of the intervention; in Active Plus50, this is enhancing and maintaining the amount of PA.

Active Plus50 focusses on influencing behavioral determinants as well as the perception of environmental determinants [26]. These are shown in Textbox 1.

Crossing the performance objectives with determinants results in change objectives. Change objectives are the intervention objectives that are specific for this intervention. The result of step 2 according to IM should be a description of the change objectives that have to be added to, altered, or removed from the original program. To optimize the change objectives to the target population, focus group interviews with the target population as well as a survey among health care professionals were conducted.

One of the theoretical frameworks that was applied in the original intervention [26] is the Theory of Planned Behavior (TPB) [30]. TPB stipulates that behavior is the result of a person’s intention, which is influenced by the following three psychological factors: attitude, subjective norm, and perceived behavioral control. Many proven effective interventions to stimulate PA were based on TPB [31]. The I-Change Model [32] is an adaptation of the TPB model. Compared with TPB, the I-Change model considers more social influences than just subjective norm, such as social support. As Active Plus65 also has a focus on the social network of participants, the I-Change model with its broader definition of social influence has been applied in this adaptation of Active Plus instead of TPB. Perceived behavioral control is called self-efficacy in I-Change but to a large degree remains the same construct. To test the opinions of the target group, the focus group questions were based on the I-Change model. Two focus group interviews with participants were conducted in January 2016. Each focus group session lasted 2 hours, and 3 researchers acted as mediators (2 PhDs in Health Psychology and 1 MSc student). The participants were recruited via local organizations for older adults, patient support groups for people with a chronic disease, a gymnastics club for older adults, and by distributing flyers in local supermarkets. A semistructured focus group interview guide was made, which contained a list of topics and open-ended questions to be discussed, based on previous evaluations of the Active Plus intervention [25], literature on behavior change strategies [32,33], and literature on the diffusion of innovations theory [34]. The focus group started with a broad opening question per topic (eg, perceived barriers) and a priori defined follow-up questions. Examples of questions that were presented to the focus group participants are as follows: “what barriers does a person as yourself with a chronic disease face, that a healthy person does not face, when being physically active?” or “what is your opinion on exercising together with other people?” Discussions were stimulated with the posing of new open questions until no new input was attained. Of both focus group interviews, audio recordings were made and transcribed verbatim. The transcripts were thematically analyzed by the first and second author, according to guidelines for content analysis of focus groups [35]. These guidelines entail (1) getting familiarized with the data, (2) generating initial codes, (3) searching for themes, (4) reviewing themes, and (5) defining and naming themes. Examples of themes were as follows: *barriers for PA when being single* and *motivation to participate in the intervention*. The findings of the first and second researcher were independently reviewed by the third researcher. After reaching agreement on the analyses, conclusions were drawn.

**Table 1:** Performance objectives (PO) of Active Plus50.

Number	Description
PO^a^ 1	Target population monitors their PA^b^ level.
PO 2	Target population indicates reasons to be physically active.
PO 3	Target population identifies solutions to remove barriers to being physically active.
PO 4	Target population decides to become more physically active.
PO 5	Target population makes specific plans to become more physically active.
PO 6	Target population increases their PA.
PO 7	Target population makes specific plans to cope with difficult situations occurring while being physically active.
PO 8	Target population maintains their PA by enhancing their routine and preventing relapses.

^a^PO: performance objectives.

^b^PA: physical activity.

**Determinants of physical activity (PA), operationalized by the performance objectives, among people older than 50 years.**

- Awareness
- Knowledge
- Commitment
- Attitude
- Self-efficacy
- Intrinsic motivation
- Intention
- Action planning
- Coping planning
- Relapse prevention skills
- Habit
- Social influence
- Perceived social environment or having an exercise partner
- Perceived physical environment

Furthermore, to optimize the match of the change objectives to the new high-risk target population, a survey among physiotherapists was conducted. It was conducted in the same municipality where the evaluation of use, appreciation, and effectiveness of the altered intervention was to be studied. By incorporating the opinion of physiotherapists, the knowledge and experience of health care experts concerning PA for older people with a chronic disease was added. Health care professionals have practical experience with the target population and may provide additional, more qualitative information than a literature study alone. This provided insights into which chronic disease needs special attention regarding PA, what type of PA is not recommendable, and how to communicate the content of advice messages. The survey was performed using a questionnaire that was sent by email. The questionnaire was sent to all 35 physiotherapists who were registered in the municipality of Heerlen, the Netherlands. The first question was to rate the necessity of a tailored PA advice for each of the most prevalent chronic diseases in the Netherlands, such as arthritis, cardiovascular pathology, lung disease, rheumatism, diabetes, cerebrovascular incidents, severe backaches, osteoporosis, and overweight [36]. The importance was rated on a scale of 1 (*not important*) to 10 (*very important*). Per the chronic disease, the physiotherapists were asked to state the three most recommendable types of PA, and the three types of PA that are not advisable.

#### Step 3: Selection of Theoretical Methods and Practical Applications

In this step, it is necessary to make sure that there are appropriate and sufficient methods for all the change objectives. For all the essential methods, it needs to be ascertained that they are properly addressed with appropriate practical applications. One of those essentials upon which Active Plus50 is based is computer tailoring. Computer tailoring is a method that assesses features, beliefs, behavior, etc, of the individual participant by using questionnaires upon which a computer program independently produces feedback. The computer program is based on if-then algorithms and deducts the appropriate advice messages from a message library. These messages are subsequently combined in a tailored advice letter. In computer tailoring, the feedback is optimally tailored to the personal characteristics of the participant. The advice messages that single older adults with a chronic disease receive, therefore, need adaptation. The effectiveness of computer tailoring in Active Plus50 has been demonstrated [27]. Apart from computer tailoring, various other theoretical methods and practical applications are used in Active Plus50. As they form the constructional base of the intervention, these have been maintained [37]. Examples of the applied theoretical methods and applications are shown in Table 2.

When the core of the change objectives remain the same, no new theoretical methods and practical applications need to be added in this step. The result of this step should be a description of the theoretical methods and practical applications that need to be added, that need to be deleted, or that must be retained.

#### Step 4: Producing Programs

In the fourth step, all the information gathered from step 1 to step 3 is combined. At this moment of the invention modification, it has been established that the program objectives and methods are appropriate. In step 4, the materials, time frame, and preferred delivery channels are analyzed. This should result in the modified intervention program.

**Table 2:** Examples of theoretical methods and practical strategies in Active Plus50.

Determinant	Theoretical method	Practical strategy	Intervention components	
			Print-delivered	Web-based
Motivation	Social modeling	Provide role model stories about intrinsic motives to be physically active.	Picture of similar individuals (same age group and gender) with quotes about their (intrinsic) motivation to be physically active	Short video of similar individuals (same age group and gender) who tell about their (intrinsic) motivation to be physically active
Awareness	Self-monitoring	Encourage monitoring of own behavior	Logbook scheme to write down their own behavior. An example was included in the advice. An empty form was attached to the advice.	Logbook scheme to write down their own behavior. An example was included in the advice. An empty form (PDF format) could be downloaded from the website.
Perceived social environment or having a sports partner	Linking members to networks of people	Provide opportunity to contact others	Post cards to invite someone to be physically active together, attached to the advice	E-cards on the website to invite someone to be physically active together
Action planning	Active learning	Invite to formulate action plans	Weekly scheme to write down plans to be physically active (when, what, where, and with whom). An example was included in the advice. An empty form was attached to the advice	Weekly scheme to write down plans to be physically active (when, what, where, and with whom). An example was included in the advice. An empty form (PDF format) could be downloaded from the website.

To achieve this goal, the existing program materials were discussed in the focus groups with the target population. Their opinions and suggestions regarding the intervention materials of Active Plus50 were identified. The first focus group was presented with the original program materials from Active Plus50; their remarks were used to adapt the program materials. The adapted materials were then presented to the second focus group, after which final adaptations were made. The materials that were presented in both focus groups were as follows: (1) the part of the questionnaire that focuses on physical impairments. As the intervention targets people with chronic diseases, it is important to get a good insight into which chronic diseases are present, and into the related impairments; (2) a bar graph showing the PA of the participant and the daily recommended PA of the Dutch population in the relevant age category. The target population, however, only consists of single people with a chronic disease, who have a lower level of PA than the general population. It was studied whether comparing the PA behavior of the new target population with the PA behavior of the general population within a bar graph was possibly perceived as unfair because chronic disease impairs PA; and (3) a modeling text. A method that is frequently used in Active Plus50 is modeling, for example, a video of a role model performing the targeted behavior. A parameter for the effectiveness of modeling is that the participant is able to identify himself with the role model. Therefore, we examined whether this identification still matched with the specific target population.

#### Step 5: Development of an Adaption and Implementation Plan

The main focus of step 5 is to plan the adoption and actual implementation of the intervention. One of the actions in this step was to ask the participants of the focus groups about their opinion on the recruitment materials of the Active Plus program. Discussed materials included a personalized letter in which people are invited to join the program and a flyer containing information about the program. The questions for the focus groups were based on the diffusion of innovations theory by Rogers [34].

According to this theory, there are several features of innovations that indicate decisively whether the innovation will actually be used. These features are relative advantage, trialability, observability, compatibility, and complexity. The relative advantage of the intervention, for example, was addressed by asking participants whether the invitation letter and flyer explained the benefits of Active Plus65 compared with existing PA programs clearly. One way in which trialability was addressed was by assessing whether the participants understood that they could participate in the program for free and could stop at any time. Whether observability was addressed properly was, among other questions, checked by asking whether the member of the focus groups expected that the results of the program would be visible among participants who had already joined the program. By asking whether there are elements in the letter or flyer that made the program sound too difficult, and that might deter them from joining the program, the perceived complexity was checked. One way in which compatibility was investigated was by asking whether the participants thought, based on the information they read in the implementation materials, that they could easily fit the program into their existing routines, and whether they disliked elements in the program.

#### Step 6: Development of Evaluation Design

In this final step, a plan was developed by which the evaluation for effectiveness and use of the altered intervention can be determined. Evaluation will progress into 2018 and contains, among other elements, conducting a longitudinal study on the level of implementation. The main outcome that will be assessed is the difference in the amount of PA between the baseline measurement and at 3 and 6 months. The relationship between social network, feelings of loneliness, and PA over time will also be assessed. Furthermore, an evaluation study will be conducted on the differences in the characteristics of the participants in relation to entry channel (Web-based or paper-based), on dropout, and on effects.

### Ethics Approval and Consent to Participate

This study was reviewed and approved by the Committee for Ethics and Consent in Research of the Open University of the Netherlands (reference number: U2016/02373/HVM). Trial registration was not applicable as this study does not report on the results of an intervention. Participants provided written informed consent to participate in the study.

## Results

In the Methods section, the theoretical processes of the IM protocol, and the way these were addressed in this research, were stated. The results and practical implications of these steps are presented in the Results section as per the individual step of the protocol.

### Step 1: Needs Assessment and Determination of Fit With the Problem

Literature shows that at older age, the majority of the people have a chronic disease. In Europe, 43% of the people in the age group of 55 to 64 years has one or more chronic diseases; this increases from 53% (64-74 years) to 64% (75-84 years) to 69% (>85 years) [38]. The most prevalent are cardiac disease, chronic pulmonary disease, and diabetes mellitus. People with a chronic disease have an enhanced risk of developing another chronic disease, that is, multimorbidity [39,40], which worsens the health situation. The prevalence of multimorbidity is increasing significantly since last decade, not only because of the aging of society but also because of changes in lifestyle risk factors, such as a lack of PA [41]. Enhancing or maintaining health is, therefore, at least equally important for the specific high-risk target population of Active Plus65 as for the original population of Active Plus50.

In the age group of 50 to 59 years, 16% of the general population report having a mobility limitation that impairs them in PA. This percentage increases from 25% (60-69 years) to 37% (70-79 years) to 61% (>80 years) [42]. In general, people with a chronic disease are less physically active than healthy people [43,44]. Chronic diseases commonly cause mobility limitations that form impairments regarding PA. People with a chronic disease may be between 1.2 and 2.7 times more likely to have a mobility disability compared with those who do not have a chronic disease [45]. Not only the mobility limitation in itself but also the fear of pain or fear of injury are related to a low level of PA for older people with a chronic disease [46,47]. The World Health Organization [1] recommends that all adults, regardless of age or the presence of a chronic disease, should perform a minimum of 150 min per week of at least moderate to intensive PA. The main behavioral target and primary aim for Active Plus65 is stimulating participants to reach a level of PA that meets this recommendation. This is the same as in the original intervention, although the point of departure with respect to PA differs substantially in the adapted target group, where all participants have one or more chronic diseases.

Apart from the limitations that are specific for older adults with a chronic disease, age in itself comes with limitations, such as impaired eyesight or hearing, which can interfere with PA [6,48-50]. In addition, here the aim with regard to PA is the same in Active Plus50 and Active Plus65; however, the target population in Active Plus65 possesses some characteristics that require a refinement of the intervention.

Another significant feature of older adults, who are less physically active, is the absence of a life partner [6,15]. Single older adults are at risk for becoming socially isolated, especially bereaved older people [51-53]. Living alone and social isolation often results in feelings of loneliness [54]. Loneliness is considered to have a significant negative influence on the physical and mental health of older adults [16,17,55]. Feelings of loneliness increase with age—the highest percentages of people who are lonely are older than 65 years [56-58]. Participating in PA, when done with others, is a way to decrease loneliness as it stimulates social contacts [59,60]. From this, the secondary goal of Active Plus65 is derived, which is stimulating PA together with other people.

The literature study, thus, reveals that the health problems and the related health behavior targeted by the original intervention (Active Plus50) are at least equally relevant for the target group in Active Plus65, which is a specific segment of the target population in the original intervention. The main aim of the original intervention, that is, to raise and maintain the level of PA, remains the same; however, an additional emphasis will be put on being physically active with others to increase the social network.

### Step 2: Logic Model of Change and Matrices

The needs assessment (step 1) has revealed that Active Plus65 aims to increase and maintain the PA of single people older than 65 years with a chronic disease. As this primary behavioral outcome of Active Plus65 is the same as for Active Plus50, the performance objectives (as previously shown in Table 1) in both interventions are identical. The secondary behavioral outcome (ie, to stimulate PA with others) is enclosed in the existing performance objectives. For example, in the second performance objective (ie, the target population can indicate reasons to be physically active), one of the reasons to be physically active could be that it enables one to engage with other people when being active with others. Another example can be found in the third performance objective (ie, the target population identifies solutions to remove barriers to being physically active); finding a stimulating partner to be physically active with can be a solution to remove a barrier.

In the Active Plus50 intervention [26], important psychological determinants have been established that need to be addressed to stimulate PA. Three major characteristics can be identified in which the target population of Active Plus65 differs from the original Active Plus50 population; these characteristics can change the impact of the established determinants. The first of these is that the entire target population of Active Plus65 has one or more chronic diseases, whereas in Active Plus50, only 45% of the participants reported having a chronic disease [27]. The number of chronic diseases, the physical limitations, and fatigue caused by the chronic disease can influence the amount of PA as well as the determinants [10,11,44,46]. The second characteristic is that the target population of Active Plus65 consists entirely of single people; in Active Plus50, only 21% of the participants were single. The social support of a partner, family, or friends as well as the available practical support they provide, such as having a sports companion, is known to influence PA [52,60,61]. The third characteristic in which the new target population differs from the original population is age; in Active Plus50, 37% of the participants were older than 65 years, whereas in Active Plus65, this will be 100%. With regard to age, experience with PA in the past, as well as perceived level of fitness and general fitness (eg, impaired eyesight, ie, not chronic diseases) may have an effect on PA [43,62,63]. The potential influence of these characteristics on PA has been endorsed by the research of Peels et al [37] on Active Plus50, which showed that especially single older people with a chronic disease expressed the need to receive more information about PA with their specific chronic disease, and the need for more personal interaction when being physically active.

Considering these characteristics, it is concluded that the determinants in Active Plus50 and Active Plus65 are identical but that the relative importance of the determinants is different. People with a chronic disease, for example, can develop a fear of PA as a result of pain avoidance beliefs [10,11,43,46]. The personal determinant attitude, under which pain avoidance beliefs can be categorized, therefore, has a relative higher importance than it has in Active Plus50.

As the performance objectives and determinants are comparable in the Active Plus50 and Active Plus65 intervention, the intervention did not require major adaptations in overall structure. The change objectives did require further refinement as the relative importance of the determinants is different. These refinements were established by the interviews with the focus groups and by the input from the physiotherapists as described below.

#### Focus Groups

The features of the 14 participants who took part in the focus groups are shown in Table 3.

By conducting focus group interviews, the opinions, wishes, and preferences of the target group were taken into consideration in the adaptation of the intervention, that is, in specifying the change objectives. Focus groups interviews were also used in the development of the original intervention [26]. According to literature, two focus groups are needed to discover the large majority of relevant themes [64]. By also including people who did not comply with the characteristics of the target group, such as married people and people without a chronic disease, potential differences of opinion between the target group and nontarget group could be identified and discussion could be stimulated. The questions for the interviews were based on the I-Change-model [32,33], and special attention was given to the characteristics that are stated above.

##### Attitude

The majority of the participants expressed that everyone can be physically active to some degree:

I exercise more now that I have a chronic impairment…I live more conscious now.

They also expressed the opinion that some people stop exercising because they experience pain because of performing their exercises in the wrong way. The reasons why the participants started exercising were diverse, such as for the exercise in itself or for the social contacts with other people during exercising:

Someone who listens to you…I really need that.

On the other hand, the participants indicated that they know people who are hesitant to join an exercise group because they do not feel comfortable to socially engage with people they do not know:

Getting out and making contact with new people…I really had to learn that again after my husband died.

##### Self-Efficacy

The participants thought that most people do not exercise because they just do not know that there are simple exercises that one can do and, therefore, do not exercise at all:

Some simple exercises that I could do in my own house would be really helpful.

**Table 3:** Features of the focus group participants.

Characteristics		Focus group 1	Focus group 2
**Gender**			
	Male, n	1	3
	Female, n	7	3
**Marital status**			
	Married, n	4	3
	Widowed, n	4	3
Mean age in years (range)		72 (62-83)	78 (70-94)
Participants with chronic disease, n		6	3

**Table 4:** Example of opinion of focus groups and resulting change in intervention.

Determinants of PA^a^	Opinion focus groups	Recommended change in intervention
Attitude	Walking and cycling are unpleasant because of busy streets	Stressing that not only outdoor activities are relevant but that performing PA at home can also be beneficial
	Future benefits are an important reason for PA	Stressing that PA is beneficial for long-term health
	Possible contacts with others are an important reason for PA	Stressing that PA can be a way to interact with others
	Lack of knowledge about PA possibilities is why people do not exercise	Adding a list of local venues and providing specific information on PA that can be done together with others
	Fear of pain deters people from PA	Encouraging people to seek advice from their physiotherapist or general practitioner so they can be reassured that the pain does not have to be harmful
Self-efficacy	Pain when exercising makes PA difficult	Adding information that pain is not necessarily bad and information on PA exercises that are less painful so people feel confident.
	Facilities not accessible for those without a car	Stressing that there are exercises to do at home, or nearby with a list of local venues
	Fear of reaction by others when joining an exercise club	Stressing that everybody feels awkward the first time but that it becomes easier soon
Social influence	Fear of going outside when it is dark	Stressing possibility to ask a friend to join them or to exercise in day time
	Lack of desire to engage in social contacts	Stressing that PA with someone else can be a pleasant way to combine sports and socializing and that after the first time, exercising in a group becomes less awkward
	Fear of initial contacts if joining an existing PA group	Using modeling to let a role model tell that they were hesitant to join a PA group but that they were pleasantly surprised by the welcome

^a^PA: physical activity.

Finding initial contacts with others is awkward when joining an exercise group for the first time; it may deter people from PA with others. About half of the participants said that a lot of venues where you can exercise are difficult to reach for older people, because you need a car to get there (and they often do not drive anymore). Some do not feel physically fit to walk alone to the exercise facility or consider their surroundings not safe enough, especially after dark:

I really do not want to get into trouble, so I stay indoors in the evenings.

##### Social Influence

The participants who had lost their partners referred to the challenges of joining social activities again after bereavement and that personal support from friends and family is essential to get on with life. They expressed that they joined an exercise group for social contacts rather than for the exercise itself. The participants also referred to people who seem to be lonely but not able to take action and socialize again:

They just don’t seem to be able to take action, no matter how much effort you put into them.

According to the participants, it is very difficult to reach those people or to stimulate them to join exercise clubs. Not having a partner also has some practical barriers, such as having no one to bring you to an exercise venue:

Going places is difficult now that I am dependent on public transport.

Table 4 provides the most relevant results of the focus group interviews.

#### Survey of Physiotherapists

Of the 35 physiotherapists who were invited to participate, 10 physiotherapists completed the questionnaire; known reasons for nonresponse were lack of time or no longer being active as physiotherapists. The physiotherapists indicated that cardiovascular problems, lung diseases, and rheumatism resulted in the largest barriers for being physically active. Cerebrovascular incidents, diabetes, severe backaches, and osteoporosis represented the second most important diseases, and overweight and arthritis the third. This information was used to determine the sequence in which advice on being physically active with a chronic disease was presented, that is, if a participant had multiple diseases, the participant only received tailored information on a maximum of three chronic diseases, and the disease for which a tailored advice is most important (ie, resulted in the largest barriers for being physically active, as defined by the physiotherapists) is presented first.

The physiotherapists were asked to give top three types of PA that were to be recommended, and PA that were to be discouraged, per chronic disease. There was a large diversity in the kind of PA that was recommended per chronic disease; this was also the case for nonrecommendable forms of PA. As reason for this diversity, all physiotherapists indicated that a standard PA advice cannot suffice because of variability of complaints within a chronic disease and degrees of severity. Moreover, the physiotherapists explained that a lot of people have more than one chronic disease, and types of PA that are beneficial to one chronic disease may be harmful to the other chronic disease. In addition, older people generally have other issues, such as poor eyesight or balance, which might impair being physically active more than the chronic disease in itself. Apart from these physical matters, the physiotherapists express the opinion that other matters can interfere with PA, such as the level of insight that people have in the seriousness of the disease, the fear of pain, and the amount of PA earlier in life. However, these matters need not to deter people from being physically active by focusing on the possibilities instead on the barriers. This feedback from the physiotherapists resulted in an increased prudence in advising participants to become more physically active; participants were, therefore, additionally advised to contact a physiotherapist or general practitioner when in doubt about their PA potential.

On the basis of the input from the focus groups with the target population and results from the survey among physiotherapists, the existing change objectives of Active Plus50 [26] were refined for the more specific high-risk target population of Active Plus65. Table 5 shows examples of existing and new change objectives.

### Step 3: Theoretical Methods and Practical Applications

No new theoretical methods and practical applications have been added, as the methods and applications used in the existing intervention already had been proven effective [27], and only needed more specific targeting to the population. Computer tailoring remained the core of the intervention; single older adults with a chronic impairment in PA received adapted advice texts, whereas participants from other subgroups received the original advice texts. The practical content, therefore, has been refined to better meet the demands of the specific target population. This is discussed in step 4.

### Step 4: Producing Programs

The questionnaire that the participants in Active Plus50 have to fill in is the core of the intervention, as it forms the input for providing tailored advice; this questionnaire was, therefore, also used in Active Plus65. All questions have been carefully reviewed to determine whether they matched the adapted change objectives.

**Table 5:** Examples of change objectives (COs) in the original and adapted intervention.

Performance objectives	Determinants			
	Attitude	Action planning	Self-efficacy	Knowledge
2. Participant indicates reasons to be physically active	Original CO^a^: participant feels positive about being sufficiently physically active			Original CO: participant knows about the health benefits of sufficient PA^b^
	Adapted CO: participant feels positive about being sufficiently physically active even if they sometimes experience pain			Adapted CO: participant knows about the health benefits of sufficient PA specifically for people with a chronic disease
3. Participant identifies solutions to remove the barriers to being physically active		Original CO: participant makes specific plans to remove barriers to being physically active	Original CO: participant feels confident about being able to take away the barriers to being physically active	Original CO: participant knows how to identify difficult situations and know manners to take away these barriers
		Adapted CO: participant makes specific plans to remove barriers to being physically active and to incorporate others into their plans	Adapted CO: participant feels confident about being able to take away the specific barriers for chronically impaired	Adapted CO: participant knows how to identify situations that are specifically difficult for single people and knows manners to take these barriers away
5. Participant makes specific plans to become more physically active	Original CO: participant feels positive about making plans to increase their PA	Original CO: participant makes specific plans to increase their PA		
	Adapted CO: participant feels positive about making plans to increase their PA and to incorporate others into their plans	Adapted CO: participant makes specific plans to increase their PA activities on their own as well as with others		

^a^CO: change objectives.

^b^PA: physical activity.

**Table 6:** Examples of changes in practical content.

Determinant	Theoretical method	Practical strategy	Practical content	
			Active Plus50	Active Plus65
Attitude	Feedback	Provide information on pros and cons	General information on benefits of PA^a^	Adding more information on being physically active with specific impairments by adding fact sheets about the recommended kind of PA per chronic condition
	Improve knowledge			Confirming that fear of pain can influence commencing PA but that there are special activities that can be done with a minimum level of pain: a specific suggestion is added: for example, a person with arthritis is advised to join a local swimming club for people with their impairment
Social influence	Facilitating	Stimulate participants to seek partners for PA	General statements in tailored advice that PA is more entertaining when done with a partner	Adding more information on local sports clubs and local patient support groups
				Adding advice that PA is a possible way to engage with other people
				Adding possibilities on the Active Plus65 website to look for a PA buddy
				Adding a brochure of social activities for older people that are organized in the municipality
				Video and pictures of people exercising alone were replaced by comparable people exercising in a group
Self-efficacy	Social modeling	Provide role model stories about difficult situations and how to cope	Picture or film of similar others (same age group and gender) with quotes about how they coped with a similar perceived difficult situation	The peers in the pictures and film were replaced by older people
				Less pictures and films with people cycling, as cycling is often too challenging for older adults, but more with people walking
				Content was added in the advice, which acknowledges that it may be awkward to join an exercise club for the first time but that these feelings diminish soon

^a^PA: physical activity.

In the questions where participants score positive and negative attributes of PA, additional items were added, based on the new change objectives. An example of a positive attribute is “PA brings me in contact with others” and an example of a negative attribute is “I avoid PA because of the pain I anticipate.” In the message library, corresponding advice messages were added. For example, those participants who avoid PA because of anticipated pain receive an advice message on coping with unavoidable pain. In addition, questions were added to determine the loneliness experienced. In the message library, corresponding advice messages were added to emphasize the positive effects of being physically active with others. Furthermore, questions were added to check whether participants used devices such as walking sticks; considering the older age of the participants in Active Plus65, tailoring to being physically active with those devices is appropriate. For these people an advice message was added to the message-library explaining that PA with these devices is possible.

For all the advice messages of Active Plus50, it was checked whether they matched the target population adequately. First, text about being physically active with a spouse, for example, was removed. Furthermore, modeling videos, pictures, and voice-overs were replaced by content with age-matched persons, that is, people older than 65 years. When making these new videos and pictures, the emphasis was put on people who were exercising with others instead of alone. In the new modeling content, mainly walking was illustrated instead of cycling as walking can generally be done better and is safer at older age than cycling.

The adequateness of the intervention materials was further studied by presenting the participants of the focus groups with the following: (1) the part of the questionnaire that addresses physical impairments; (2) a bar graph showing the PA of the participant, the average PA in their age category, and the daily recommended PA; and (3) a modeling text. Focus group participants were asked to fill in the questionnaire to detect problems in the intervention or tailoring questionnaire. Half of the participants in the focus groups experienced difficulties while filling in the questionnaire, resulting in the redesign of this part of the questionnaire.

**Table 7:** Examples of feedback from focus groups and modifications to original Active Plus program.

Results focus group	Objective or strategy	Modifications
Introduction letter is not clear about Web-based and printed possibilities	Raising participation levels by giving people a choice in the delivery method of the intervention	Rewriting the introduction letter so that the choice between Web-based and printed is clearer
Identity of sender of introduction letter is not clear	Emphasizing importance of the intervention by making clear that the sender is the municipality	Having the letter signed by the principal council member of the local municipality
Kick-off meeting is considered to be interesting	Giving information about the program and giving opportunity to meet others	Organizing a kick-off meeting for potential participants

The bar graph was shown and the focus groups were questioned to check whether they could interpret the bar graph correctly. The majority of the participants were not able to do so; therefore, a written explanation was added to the bar graph. The modeling text was also presented to the focus groups. Four participants found the text inspiring. One participant interpreted the text that PA could cure a chronic disease; therefore, the text was refined. In Table 6, examples are given of how the comments from the focus groups were used to adapt the practical content of Active Plus65.

### Step 5: Implementation

During the focus groups, participants were presented with the following recruitment materials for the program: (1) a personalized letter in which people are invited to join the program, (2) a flyer containing information about the program, and (3) information on a kick-off meeting. The flyer was considered to be clear and motivating, but all participants found the letter confusing and not arousing interest in the program. After the first focus group, the letter was altered and presented to the second focus group. In this group, the letter was considered to be clear and motivating. The participants were also asked whether they would participate in a kick-off meeting of the program where they could receive more information on the program and meet other participants; this produced positive reactions. In Table 7, examples of the alterations in implementation materials after conducting the focus groups are given.

### Step 6: Planning Evaluation

To complete the systematic adaptation, a plan for the evaluation of the intervention was developed. For this, a longitudinal study will be conducted in one Dutch municipality. All citizens who are registered as single and are 65 years or older will be invited to participate in Active Plus65 (N=6751). The invitation letter will make it clear that the program is especially targeted at single older people with a chronic disease.

The primary outcome is the difference in PA between baseline and 3 and 6 months. The amount of social contacts and perceived feelings of loneliness will also be measured. Furthermore, possible moderators or mediators of the results on the primary outcome will be assessed, such as age. To compare the effects on PA of the altered intervention with the proven effective intervention, data on participants in Active Plus50 will serve as a reference group. The evaluation of the intervention will also focus on the relationship between the demographics of the participants of Active Plus65 and characteristics such as participation in the Web-based and paper version as well as attrition ratios.

## Discussion

### Principal Findings

The aim of this study was to describe the systematic development of a computer-tailored eHealth intervention aimed at increasing PA for single people older than 65 years who have a chronic disease. This intervention, Active Plus65, was developed by adapting the proven effective PA intervention, Active Plus50 (for the general population older than 50 years), to better fit the abovementioned narrower target population.

It is concluded that even when the new target population is a sizable segment of the original target population, the original proven effective intervention may not optimally fit the different subpopulations. Therefore, the original intervention needed enhancement to achieve this. The necessary adaptations were performed in a systematic way by using the IM protocol [29] to ensure that both theory and empirical evidence are encased in the intervention. The intervention will be evaluated in a longitudinal study by comparing the adapted intervention with the original intervention.

### Methodological Issues

As adapting interventions in a systematically planned way increases the likelihood of effectiveness, the use of the IM protocol increases the chance that the adapted intervention will still demonstrate effectiveness. It enables the developer to retain the components that are crucial to the effectiveness by carefully considering the elements that can be modified without lowering the effectiveness substantially. Another strength of this study is that it combines data gathered from existing research, from focus groups, as well as from health care professionals. Different angles and interests could, therefore, be incorporated; limitations when using each of these research methods separately are thus overcome.

However, there are some limitations for the approach we used within this research that need to be considered. First of all, selection bias in the focus groups might have occurred as those who participated may not have been the most optimal representatives for the target population. The majority (57%) of participants in the focus groups consisted of the members of a gym club who are possibly more physically active than others of their age and more willing to participate in joined activities. However, 64% of them had one or more chronic diseases by which representativeness issues were balanced. By adding information from a literature study and from health care professionals, potential representativeness issues were also overcome. The latter have an expert opinion on PA for chronically ill older adults and, moreover, have practical experience with their PA behavior. Furthermore, as the main goal of the intervention is to raise the level of PA, preferably together with others, the opinion of people who already are physically active is of major interest.

Another matter to consider is that recommendations for the advised amount of PA for people with a chronic disease are not totally clear yet because of a lack of scientific evidence. For this reason, the focus in Active Plus65 is put mainly on increasing PA, and not on achieving an advised amount. The planned effect evaluation will, therefore, not be able to compare the effects with a generally accepted norm.

Furthermore, there are some practical matters to consider when applying the IM protocol to adapt an existing intervention. First of all, even when not designing a new intervention, but merely adapting an existing intervention, IM is a process that is time-consuming, which should be taken into account in planning human resources and applying for funds. Although we have been able to systematically follow the IM procedure completely, a lack of time or resources could potentially result in researchers skipping elements of IM. Second, another challenge was to adequately limit the information that was added to the intervention for the specific target population to prevent an overload of advice. By adding information on being physically active with others or on being physically active with a chronic impairment, a constant balance had to be made between what to add and what could possibly be deleted. The IM protocol for adapting interventions does not specifically seem to address the issue of potential information overload. Third, although IM describes the general tasks for each step of adapting an intervention, a practical elaboration on how these matters can be addressed is lacking. Adding this information to the protocol might form a practical and valuable guideline for researchers.

### Conclusions and Further Research

Notwithstanding the abovementioned limitations, it is concluded that this paper provides valuable information for the process of adapting lifestyle interventions. The next phase in the adaptation process is a pilot testing of the adapted intervention, which may result in further refinement. After this, a longitudinal study of the implementation results will be conducted. If the effects are similar to the original Active Plus50 program, which has been proven to be effective, Active Plus65 will be implemented on a broader scale.

## Abbreviations

PA: physical activity
IM: Intervention Mapping
PO: performance objective
CO: change objective
TPB: theory of planned behavior
